# Muscle–Nerve Signaling and Neurogenic Inflammation in Temporomandibular Disorders: Potential Contributions of Occlusal Interference and Other Peripheral Triggers

**DOI:** 10.3390/dj14080495

**Published:** 2026-08-07

**Authors:** Yi Xia, Jingze Lu, Xingmei Feng

**Affiliations:** 1 School of Medicine, Nantong University, Nantong 226001, China; xiayi@stmail.ntu.edu.cn (Y.X.); lujingze@stmail.ntu.edu.cn (J.L.); 2 Department of Stomatology, Affiliated Hospital of Nantong University, Nantong 226001, China

**Keywords:** occlusal interference, temporomandibular joint, neurogenic inflammation, muscle–nerve signaling, pain mechanisms

## Abstract

Temporomandibular disorders (TMD) comprise a heterogeneous group of pain and dysfunction conditions involving the temporomandibular joint, masticatory muscles, and related structures. Although occlusal interference has long been discussed in relation to TMD, current evidence does not support an occlusion-centered etiological model for most patients. Within the contemporary biopsychosocial framework embodied by the Diagnostic Criteria for Temporomandibular Disorders (DC/TMD), occlusal interference is better regarded as one of several potential peripheral biomechanical inputs that may interact with individual pain susceptibility, parafunctional loading, inflammatory status, and psychosocial factors. This review synthesizes evidence on how peripheral biomechanical and inflammatory inputs may engage masticatory muscle–nerve signaling, trigeminal nociceptor activation, and neurogenic inflammation. We discuss altered masticatory muscle activity, proprioceptive and nociceptive afferent signaling, neuropeptide release, neurovascular and mast cell–nerve interactions, and glial activation within trigeminal pain pathways. Molecular mechanisms, including TRP channel and P2X3 receptor activation, voltage-gated ion channel dysregulation, MAPK, PI3K/Akt/mTOR, cAMP/PKA/CREB signaling, and epigenetic regulation, are reviewed as candidate pathways linking peripheral input to pain-related plasticity. These mechanisms are further considered in relation to hyperalgesia, mechanical allodynia, pain memory, emotional and cognitive modulation, and sex-related differences in pain processing. Finally, we evaluate translational implications, including mechanism-oriented animal models, exploratory biomarkers, human-derived experimental systems, and mechanism-informed interventions, while emphasizing their current limitations. Overall, this review proposes a cautious mechanistic framework in which peripheral inputs may contribute to TMD-related pain amplification in selected contexts, but clinical translation requires validated phenotyping, longitudinal evidence, and integration with conservative, reversible, and patient-centered standard-of-care management.

## 1. Introduction

TMD constitute a heterogeneous group of conditions affecting the TMJ, masticatory muscles, and associated structures and represent a substantial global health burden, with a prevalence estimated at 5–12% [1]. However, this estimate should be interpreted in context, as reported prevalence varies considerably according to population characteristics, diagnostic criteria, case definitions, and whether pain-related or broader TMD categories are included [2,3]. Characterized by pain, functional limitation, and reduced quality of life, TMD pose significant clinical challenges due to their multifactorial etiology and incompletely defined mechanistic underpinnings [4].

Contemporary TMD research and clinical practice are increasingly grounded in standardized diagnostic and biopsychosocial frameworks. This reflects a broader conceptual shift in the field, from earlier occlusion-centered models toward a multidimensional understanding of TMD that emphasizes pain mechanisms, patient-centered assessment, and conservative management [4,5]. DC/TMD provides an internationally accepted system for classifying common TMD conditions through Axis I clinical diagnoses, while Axis II instruments assess pain intensity, pain-related disability, jaw functional limitation, parafunctional behaviors, and psychosocial factors [6]. Large prospective studies, including the OPPERA program, further support the view that painful TMD arise from interacting biological, psychological, genetic, behavioral, and environmental factors rather than from a single local cause [7]. Therefore, any mechanistic discussion of TMD should distinguish between clinical causality, disease association, and experimentally supported biological plausibility.

Among the proposed peripheral contributors, occlusal interference has historically been discussed in relation to the initiation or persistence of TMD; however, its role remains controversial. Current evidence does not support occlusal interference or dental occlusion as a primary etiological factor for most TMD patients, and systematic evaluations have reported that associations between occlusal features and TMD are generally weak, inconsistent, or clinically limited [8]. Recent standard-of-care recommendations also emphasize that TMD management should be framed within a multifactorial biopsychosocial model rather than an occlusion-centered etiological model [9]. Accordingly, in this review, occlusal interference is not considered a direct or sufficient cause of TMD. Instead, it is discussed as one of several potential peripheral biomechanical inputs that, in selected clinical contexts or experimental models, may interact with masticatory muscle stress, trigeminal nociceptive signaling, and pain sensitization.

Within this more cautious framework, neurogenic inflammation provides a biologically plausible mechanism by which peripheral mechanical inputs may be translated into persistent pain amplification. Neurogenic inflammation involves activation of trigeminal sensory afferents and the release of neuropeptides, particularly CGRP and substance P, which can modulate vascular and immune responses [10,11,12,13,14]. Persistent trigeminal nociceptive and neuroimmune signaling may subsequently contribute to peripheral and central sensitization [15,16,17,18]. The bidirectional crosstalk between masticatory muscle activity and neural signaling may therefore represent a relevant pathway through which local biomechanical inputs contribute to sustained nociceptive signaling, particularly in patients with coexisting myofascial pain, parafunctional loading, or increased pain sensitivity.

At the molecular level, mechanosensitive ion channels and inflammatory signaling pathways have been increasingly recognized as important mediators linking mechanical stimulation to nociceptive and inflammatory responses. In particular, transient receptor potential channels such as TRPV4 have been implicated in mechanosensory and nociceptive signaling, and sensory-neuron TRPV4 has been shown to modulate CGRP-dependent TMD-related pain in mice [19]. Experimental models of occlusal alteration or TMJ inflammation suggest that modulation of these pathways can attenuate synovial inflammation, disk degeneration, and pain-related behaviors. Nevertheless, such findings should be interpreted as mechanistic evidence rather than direct proof of clinical causality. The precise cascade linking occlusal perturbation to muscle–nerve signal transduction, neurogenic inflammation, and central sensitization remains incompletely defined.

**Figure 1:**
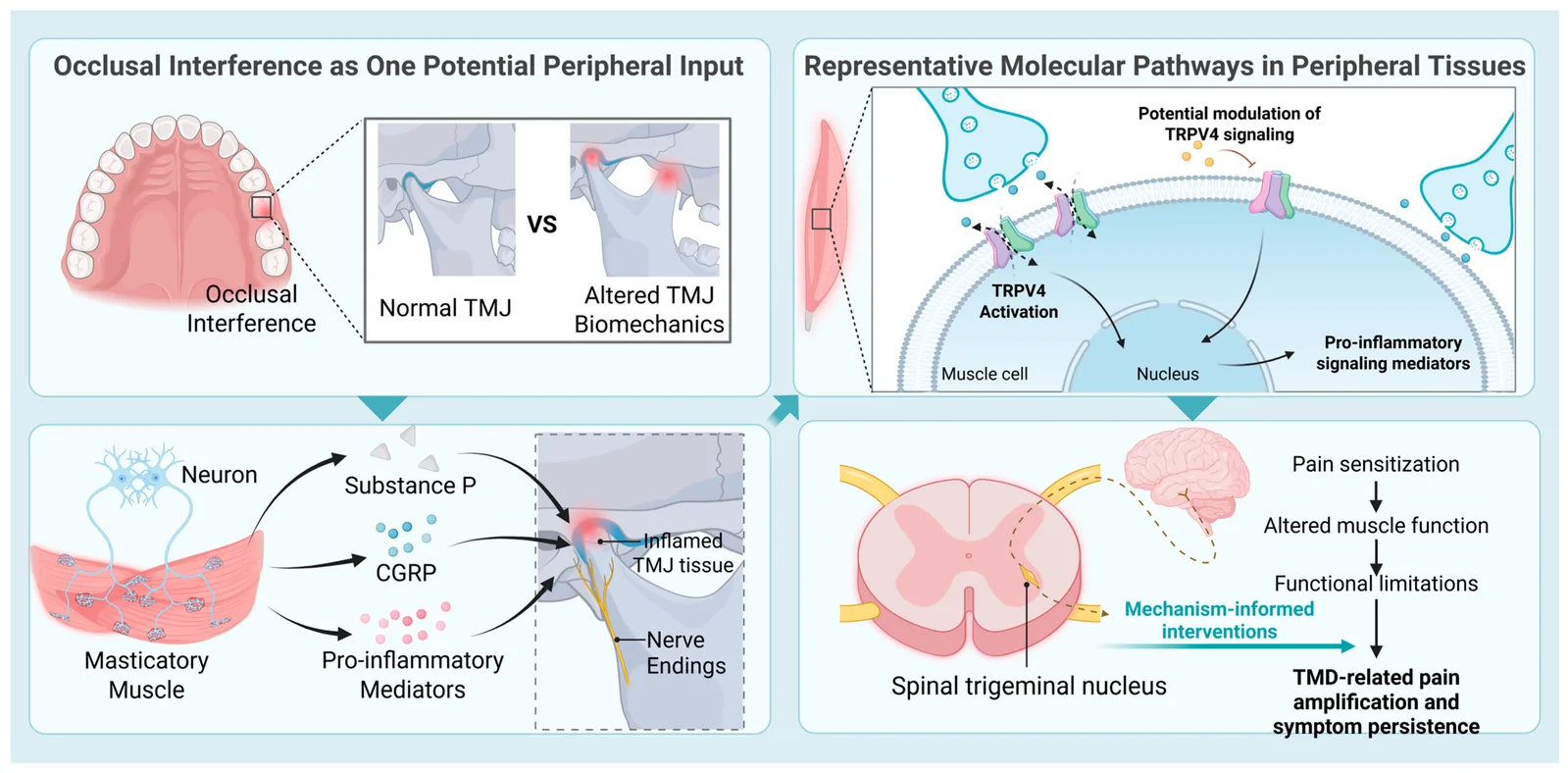
Proposed framework linking peripheral biomechanical inputs, muscle–nerve signaling, neurogenic inflammation, and pain sensitization in TMD. Occlusal interference is shown as one potential peripheral input that may interact with altered TMJ biomechanics, masticatory muscle activity, trigeminal nociceptive signaling, and neurovascular inflammatory responses. This schematic emphasizes biological plausibility within a multifactorial TMD framework rather than direct clinical causality.

Given these gaps, a balanced synthesis of current evidence is warranted. This review does not seek to re-establish occlusal interference as a primary etiological determinant of TMD. Instead, it examines how peripheral biomechanical inputs, including occlusal interference in selected contexts, may contribute to muscle–nerve signaling, neurogenic inflammation, and pain sensitization. By distinguishing among clinical, animal, and in vitro evidence, this review aims to provide a mechanistic model within the contemporary DC/TMD-based and biopsychosocial framework, while avoiding overextension from experimental plausibility to clinical causality (Figure 1).

## 2. Occlusal Interference as a Peripheral Biomechanical Input and Masticatory Muscle–Nerve Responses

Before discussing occlusal interference-related biological responses, it is necessary to clarify the scope of this concept. Clinically, occlusal interference generally refers to premature, undesirable, or non-functional tooth contacts that may disturb stable occlusal contact or mandibular movement [20]. This concept should be distinguished from broader forms of mechanical overload, such as bruxism, clenching, or repetitive jaw loading [21], and from experimental occlusal trauma models in which resin elevations, bonded crowns, artificial crowns, or dental modifications are used to impose controlled biomechanical perturbations [22,23]. Evidence from such models is valuable for exploring biological plausibility, but it should not be interpreted as direct proof that clinical occlusal interference is a primary etiological factor for TMD [8].

### 2.1. Mechanical Loading of the Masticatory Muscles and Afferent Signaling

Occlusal interference may alter condylar position and mandibular movement trajectories and may thereby modify the mechanical demands placed on the masticatory system [24]. Under selected conditions, these changes may modify the recruitment patterns of jaw-closing muscles, particularly the masseter and temporalis muscles, thereby generating local mechanical stress responses. In skeletal muscle, repetitive or excessive loading has been associated with micro-injury, impaired perfusion, local metabolic stress, delayed recovery, fatigue, and pain [25,26]. However, these effects should be interpreted as loading-related muscle responses rather than as evidence that occlusal interference alone causes TMD.

Altered mechanical loading may also influence proprioceptive and nociceptive signaling within the masticatory system. Muscle spindles and Golgi tendon organs (GTOs), which are present in several jaw-closing muscles and provide proprioceptive feedback, may exhibit altered afferent signaling when masticatory muscle recruitment or functional demand is modified [27]. In parallel, repeated activation of muscle nociceptors may contribute to hyperalgesia, allodynia, and pain amplification, particularly in individuals with pre-existing pain sensitivity or inflammatory conditions [28]. Experimental occlusal alteration and craniofacial myalgia models support the biological plausibility that sustained or sensitized masticatory muscle input can engage trigeminal nociceptive pathways [29,30]. Nevertheless, these models often involve resin elevations, bonded crowns, dental modifications, neurotrophic sensitization, or other controlled perturbations that are more intense or artificial than typical clinical occlusal interference. Their translational relevance should therefore be interpreted cautiously.

Taken together, peripheral biomechanical inputs, including occlusal interference in selected circumstances, may modulate masticatory muscle activity, proprioceptive feedback, and nociceptive afferent signaling. These processes may contribute to symptom amplification in susceptible individuals, particularly when combined with parafunctional activity, psychological stress, pre-existing pain sensitivity, or inflammatory joint conditions. Accordingly, occlusal findings should be interpreted within a comprehensive TMD assessment rather than as isolated determinants of disease onset.

### 2.2. Potential Adaptive Responses of Masticatory Muscles to Altered Loading

The relationship between altered occlusal loading and adaptive changes in masticatory muscle fibers remains an important but incompletely defined area of investigation. One proposed adaptation is the transformation of muscle fiber types, particularly a shift between slow-twitch and fast-twitch fiber profiles under altered functional demand [31,32,33]. Such changes may reflect neuromuscular adaptation to increased or asymmetric loading, especially in the masseter and temporalis muscles. However, because the evidence linking clinical occlusal interference specifically to fiber-type transformation is limited, this mechanism should be presented as a potential response to altered loading rather than a consistently demonstrated feature of occlusion-related TMD.

In addition to fiber-type adaptation, prolonged masticatory muscle overactivity may be associated with muscle fatigue, tenderness, and myofascial trigger points. Trigger points are hyperirritable regions within taut bands of skeletal muscle and may contribute to local and referred pain in myofascial TMD [34,35]. Nevertheless, trigger point formation is multifactorial and may be influenced by parafunctional activity, sustained contraction, psychological stress, sleep disturbance, and central pain modulation rather than by occlusal interference alone [34,36]. Therefore, when trigger points are discussed in relation to occlusal interference, they should be framed as part of a broader myofascial pain mechanism.

Altered loading may also influence local hemodynamics and energy metabolism within the masticatory muscles. Reduced blood flow, local ischemia, and accumulation of metabolic byproducts have been proposed as contributors to muscle pain and fatigue [25,37,38]. However, these mechanisms are better supported in the broader literature on myofascial pain and muscle overuse than as direct consequences of clinical occlusal interference. Thus, hemodynamic and metabolic changes should be interpreted as possible downstream effects of sustained muscle overactivity or mechanical overload, requiring more direct evidence in TMD-specific and occlusion-related models [39].

In summary, adaptive responses of the masticatory muscles may include changes in muscle activity, fatigue, tenderness, trigger point-related pain, local metabolic stress, and possibly fiber-type remodeling. These responses may initially represent compensatory adaptation to altered functional demand, but in susceptible individuals, they may contribute to persistent nociceptive input and pain amplification. Clinically, these findings support careful evaluation of masticatory muscle function, parafunctional behaviors, pain sensitivity, and psychosocial context.

### 2.3. From Peripheral Muscle Inputs to Pain Sensitization

Persistent or repeated peripheral input from masticatory muscles may contribute to the transition from local muscle pain to broader pain sensitization [40,41]. Potential mechanisms include sustained activation of muscle afferents, altered sensory processing, neuroimmune signaling, and plastic changes within trigeminal and higher-order pain pathways. Recent anatomical and clinical studies support the biological plausibility of this framework by showing complex sensory innervation of masticatory muscles and associations among central sensitization, somatization, and chronic masticatory muscle pain [42]. Experimentally imposed occlusal alterations may also be associated with structural and functional jaw-muscle changes, including injury responses, limited fibrosis-like adaptation, myogenesis, masseter atrophy, and altered muscle-fiber phenotype [43,44]. However, the extent to which these processes occur specifically in response to clinical occlusal interference remains insufficiently established.

Central sensitization represents a key mechanism through which persistent peripheral input can lead to amplified pain perception. It is characterized by increased excitability of central nociceptive neurons, reduced pain thresholds, enhanced temporal summation, and pain responses that may persist even after the initial peripheral stimulus has diminished. Patients with TMD often exhibit widespread pressure pain sensitivity and other findings consistent with central sensitization [45]. Therefore, occlusal or masticatory muscle inputs, when present, should be viewed as potential contributors to a broader sensitization process rather than as the sole drivers of chronic pain.

**Figure 2:**
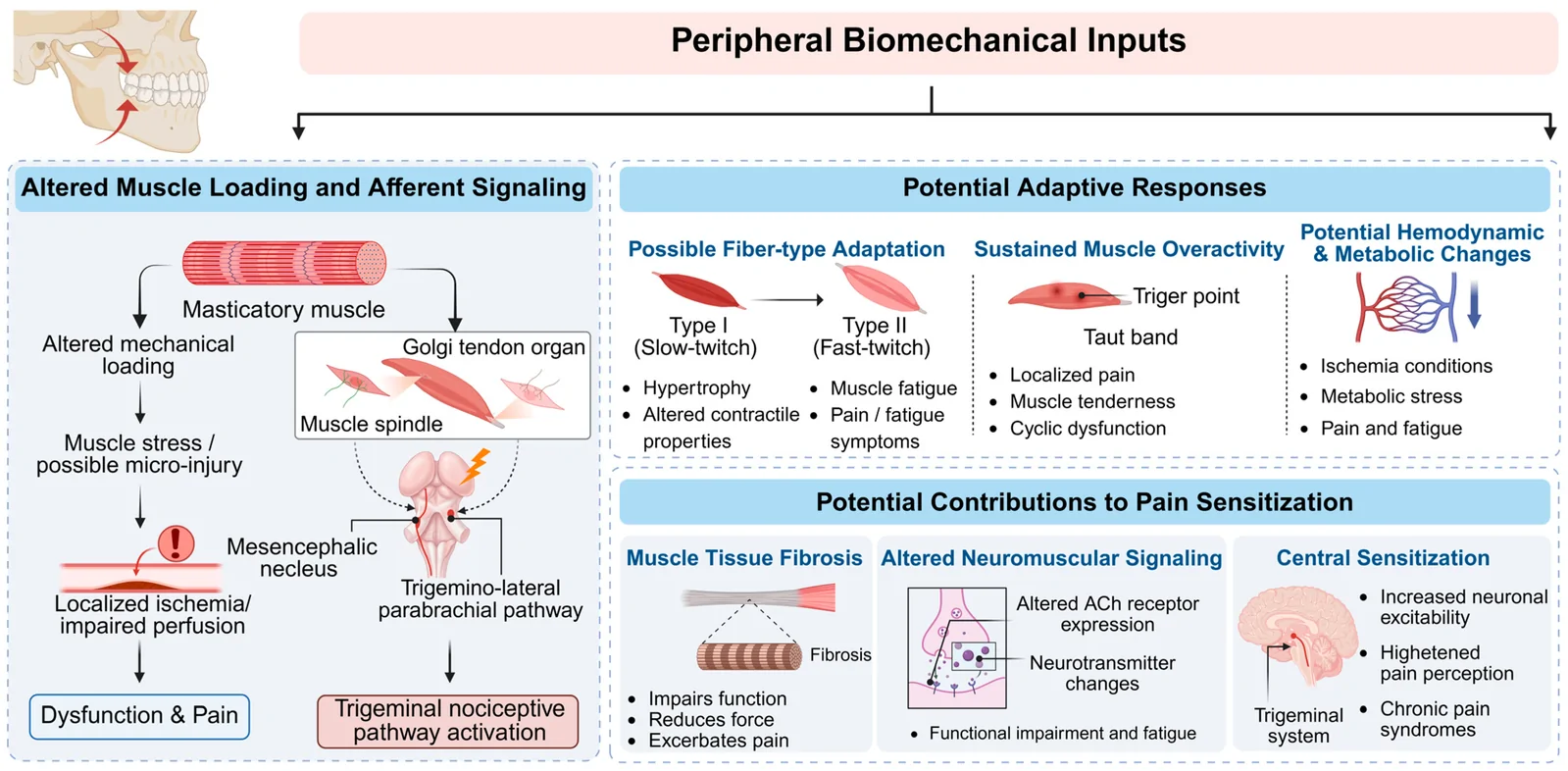
Potential effects of peripheral biomechanical inputs on the masticatory system. Altered muscle loading may influence proprioceptive and nociceptive afferent signaling, muscle stress, adaptive muscle responses, local hemodynamic and metabolic changes, and trigeminal nociceptive pathway activation. These processes may contribute to pain sensitization and symptom amplification in susceptible individuals. The lightning symbol indicates trigeminal nociceptive pathway activation, and the downward arrow indicates reduced local perfusion or impaired hemodynamic conditions.

The cumulative effects of altered biomechanical input on the masticatory system are therefore best understood within a biopsychosocial and pain-sensitization framework. Peripheral muscle changes, altered afferent signaling, central sensitization, psychosocial stress, and individual pain susceptibility may interact to shape the clinical presentation of TMD [6,42,46]. Further research is needed to determine which patients, under which conditions, and through which mechanisms occlusal interference may meaningfully contribute to TMD-related pain. Such an approach may help refine patient stratification and guide mechanism-informed but clinically cautious management strategies [47] (Figure 2).

## 3. Neurogenic Inflammation and Trigeminal Nociceptive Signaling

Neurogenic inflammation represents a cascade of events through which peripheral nociceptor activation can be translated into local inflammatory amplification and, under persistent conditions, pain sensitization. In TMD-related pain, particularly when peripheral biomechanical or inflammatory inputs are present, this process may involve three interconnected components: activation of trigeminal nociceptors, neuropeptide-mediated neurovascular and immune interactions, and glial cell-mediated amplification within trigeminal pain pathways. This section discusses these mechanisms as biologically plausible pathways linking peripheral input to pain amplification.

### 3.1. Activation of Peripheral Nociceptors

The activation and sensitization of peripheral nociceptors are critical events in the pathophysiology of pain, including TMD-related pain. Trigeminal ganglion neurons innervating the TMJ and masticatory muscles express multiple receptors and ion channels that can respond to inflammatory and mechanical stimuli. Among these, transient receptor potential vanilloid 1 (TRPV1), transient receptor potential vanilloid 4 (TRPV4), and purinergic P2X3 receptors have received particular attention in orofacial pain research. TRPV1 is classically activated by noxious heat, protons, and capsaicin, whereas P2X3 receptors are activated by ATP released during tissue injury and inflammation. Experimental studies have shown that P2X3 receptor expression can increase in trigeminal ganglion neurons in models of TMJ inflammation or occlusal interference, supporting its potential role in orofacial mechanical hypersensitivity [48,49]. Functional interaction between P2X3 and TRPV1 in trigeminal sensory neurons further suggests that these channels may cooperate in peripheral sensitization [50]. In addition, TRPV4 has been implicated in TMJ inflammation-evoked pain behavior and trigeminal ganglion nociceptive changes [51]. These findings support the plausibility that peripheral biomechanical or inflammatory inputs may sensitize trigeminal nociceptors, but they should be interpreted as mechanistic evidence rather than direct proof of clinical causality.

Importantly, activated nociceptors do not merely transmit pain signals; they can also contribute to the local inflammatory milieu through neuropeptide release. CGRP and substance P are among the major neuropeptides released from activated sensory afferents and are central mediators of neurogenic inflammation [10,52]. CGRP, in particular, can induce vasodilation and plasma extravasation, thereby linking nociceptive activity to vascular and inflammatory responses [11,12,15]. Beyond vascular effects, CGRP may modulate immune cell functions, including macrophage polarization and dendritic cell/T-cell activity, indicating a broader role in neuroimmune regulation; however, the evidence regarding dendritic cells and T cells primarily comes from general immunology studies and requires further validation through specific TMD research [13,14,53].

The interaction between nociceptors and immune cells further contributes to peripheral sensitization. Nociceptors can influence immune cell activation and recruitment, whereas inflammatory mediators such as cytokines, prostaglandins, ATP, and neurotrophic factors can further sensitize nociceptors, creating a feed-forward loop that amplifies pain signaling [54,55]. In TMD, such neuroimmune crosstalk may be particularly relevant when local TMJ inflammation, masticatory muscle overactivity, parafunctional loading, or increased pain sensitivity coexist.

### 3.2. Neurovascular and Mast Cell–Nerve Interactions

Once activated, peripheral nociceptors can trigger neurovascular events that convert local neural activity into inflammatory amplification. CGRP-mediated vasodilation is a well-established component of neurogenic inflammation [10], and nitric oxide (NO) may further participate in vascular signaling and vasodilation-related responses, as suggested by iNOS expression in diseased TMJ synovial tissues, NO metabolites in synovial fluid, and recent TMJ-OA evidence linking iNOS-related signaling to macrophage-mediated synovial inflammation and cartilage degradation [56,57,58]. However, direct evidence that NO-mediated vasodilation is a major driver of clinical TMD symptoms remains limited. These vascular events can increase local blood flow and vascular permeability, facilitating the entry of plasma proteins and immune cells into the affected tissue. In the TMJ and surrounding tissues, such mechanisms may contribute to peripheral inflammatory amplification when local nociceptive or inflammatory stimuli persist.

Mast cells represent another important interface between sensory nerves, blood vessels, and immune responses. Mast cells can be activated by neuropeptides and inflammatory mediators, leading to the release of histamine, cytokines, proteases, and other vasoactive substances. These mediators can increase vascular permeability and sensitize nearby nociceptive endings, thereby reinforcing neurogenic inflammation [59,60]. Conversely, sensory nerve-derived neuropeptides such as substance P and CGRP may influence mast cell activation, forming a bidirectional mast cell–nerve interaction. Evidence of neuropeptide-containing nerves and mast cells in human TMJ tissues further supports the relevance of this interaction to the temporomandibular region [61].

Beyond neuropeptide-mediated vascular responses, VEGF may also contribute to neurovascular remodeling and inflammation in TMJ disorders. VEGF expression in dysfunctional TMJ disks suggests its role in local tissue remodeling and vascular responses [62]. In synovial tissues from patients with TMJ internal derangement, the expression of VEGF, fibroblast growth factor-2, and their receptors has been correlated with angiogenesis, further supporting a role for VEGF-related signaling in pathological TMJ vascular remodeling [63]. Elevated VEGF levels in synovial fluid from patients with symptomatic TMJ internal derangement further suggest local inflammatory or angiogenic activity within the joint space [64]. Mechanistically, VEGF, also known as vascular permeability factor, can promote microvascular hyperpermeability and angiogenesis, thereby facilitating inflammatory mediator extravasation and tissue remodeling [65]. Although direct evidence linking VEGF to trigeminal neurogenic inflammation in TMD remains limited, these findings suggest that VEGF may act as a TMJ-associated neurovascular modulator connecting local inflammation, vascular remodeling, and pain amplification in the trigeminal system.

In summary, neurovascular and mast cell–nerve interactions provide a plausible pathway by which trigeminal nociceptor activation may be amplified into local inflammatory responses and peripheral sensitization. These mechanisms may contribute to TMD-related pain amplification, particularly in inflammatory or myofascial phenotypes, but their direct relationship to clinical occlusal interference requires further investigation.

### 3.3. Glial Cell Activation and Trigeminal Pain Amplification

While peripheral nociceptor activation and neurovascular interactions may initiate and amplify local neurogenic inflammation, the transition from transient peripheral input to persistent pain may be influenced by glial cell activation within trigeminal pain pathways. Glial responses can occur both in peripheral sensory ganglia, particularly the trigeminal ganglion, and in central structures such as the spinal trigeminal nucleus. Experimental TMJ inflammation activates satellite glial cells and resident immune cells in the trigeminal ganglion and microglia in the spinal trigeminal nucleus [16]. These responses are important for understanding how peripheral input may be transformed into sustained peripheral and central sensitization.

Satellite glial cells, which are critical for the homeostasis of sensory neurons, exhibit notable proliferation and phenotypic changes in response to inflammatory stimuli. Studies suggest that in trigeminal pain conditions, satellite glial cells may shift from a mainly supportive role toward an active modulatory role in neuronal excitability and pain signaling [66]. For example, in the trigeminal ganglion, these cells may proliferate and undergo phenotypic changes, including increased GFAP expression, which is linked to neuroinflammation and pain modulation [67]. This activation may promote the release of pro-inflammatory cytokines (e.g., IL-1β, TNF-α) [67,68] and other neuroactive mediators that further influence neuronal excitability and pain perception [17]. The interplay between satellite glial cells and sensory neurons is particularly evident in inflammatory and neuropathic orofacial pain, where glial activation contributes to the sensitization of pain pathways.

Extending from the periphery to the central nervous system, microglia, the resident immune cells of the central nervous system, can also amplify neuroinflammation and central sensitization. In trigeminal pain pathways, glial involvement has been demonstrated in the spinal trigeminal nucleus, where glial activation can facilitate central sensitization after peripheral inflammation or injury [69]. Upon activation, microglia can release a variety of pro-inflammatory cytokines and neuromodulators that exacerbate neuroinflammation and contribute to the pathogenesis of pain syndromes [18]. This signaling cascade not only amplifies the inflammatory response but may also shift microglia toward a pro-inflammatory and pain-facilitating state.

Connexin-mediated intercellular communication may provide an additional mechanism for neuron–glia interactions within the trigeminal ganglion. Experimental evidence indicates that connexin expression is differentially regulated in response to TMJ inflammation. In one study, acute and chronic TMJ inflammation increased the expression of Cx26, Cx36, and Cx40 in trigeminal ganglion neurons and/or satellite glial cells, whereas Cx43 expression was not significantly increased [70]. However, subsequent studies using different TMJ inflammatory pain models reported increased Cx43 expression in satellite glial cells or trigeminal ganglion tissue and demonstrated that inhibition of Cx43 reduced inflammation-induced hypernociception and TMJ-evoked jaw-muscle responses [71,72]. These findings highlight the dynamic and context-dependent role of connexin-mediated satellite glial cell–neuron communication in trigeminal neuroinflammation, which may support neuronal homeostasis under physiological conditions but contribute to neuronal hyperexcitability and pain amplification during persistent inflammation.

**Figure 3:**
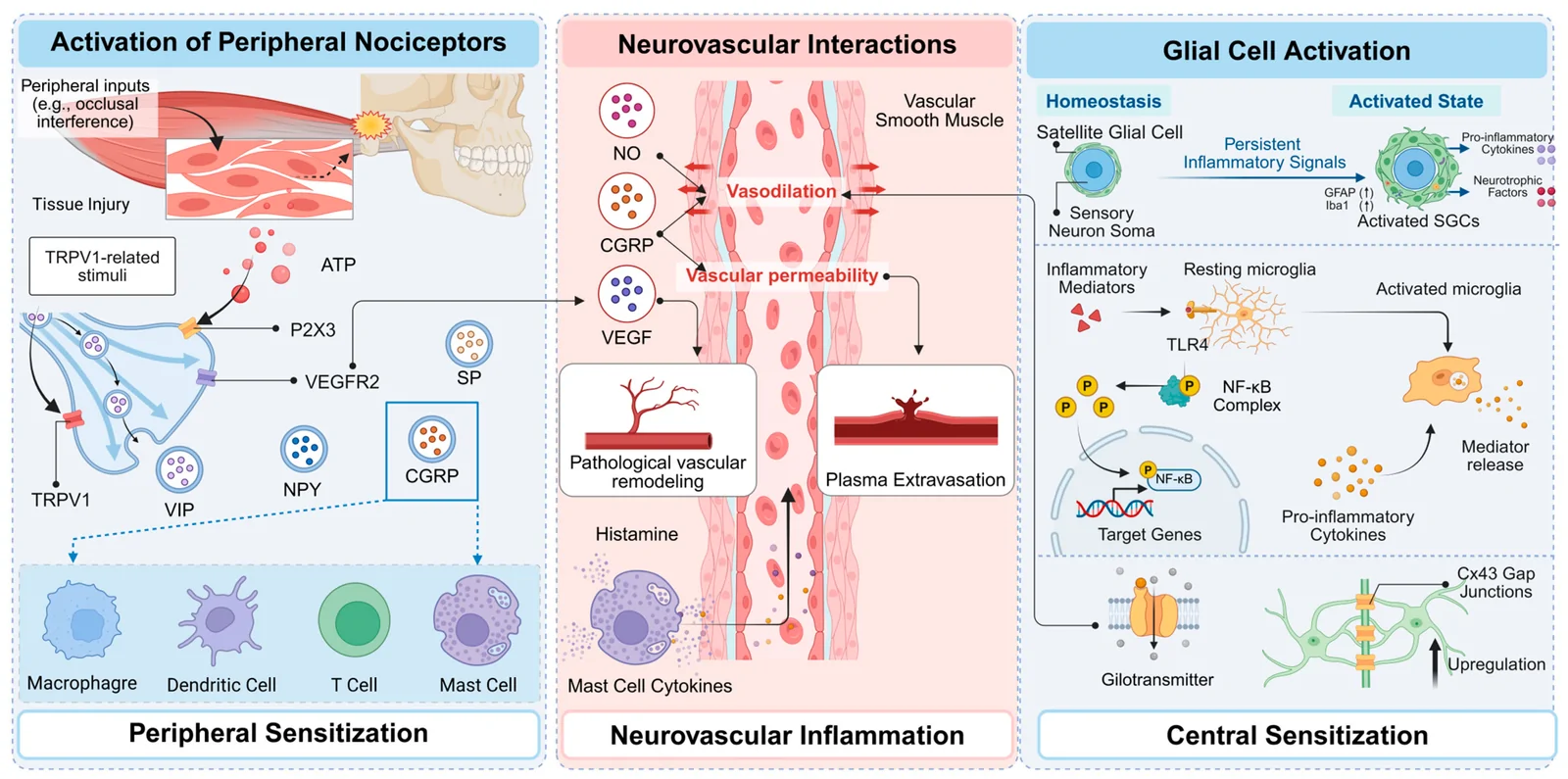
Potential neurogenic inflammatory pathways linking peripheral inputs to trigeminal pain amplification. Activation of peripheral nociceptors may promote neuropeptide release, immune cell modulation, neurovascular responses, mast cell–nerve interactions, and glial activation. These interconnected mechanisms may support peripheral and central sensitization in TMD-related pain. The red bidirectional arrows indicate vasodilation during neurovascular responses. The arrows in the glial activation panel indicate phenotypic transition toward activated glial states and increased regulation of connexin-mediated intercellular communication.

Taken together, glial–neuronal interactions highlight the complexity of neuroinflammatory responses and suggest that glial signaling may represent a potential therapeutic target in TMD and other chronic pain conditions (Figure 3).

## 4. Molecular Mechanisms and Signal Transduction

### 4.1. Ion Channel Regulation

As described in Section 3.1, peripheral biomechanical or inflammatory inputs may activate and sensitize trigeminal nociceptors through receptors and ion channels such as TRPV1, TRPV4, and P2X3. These functional changes are fundamentally governed by the regulation of ion channels that control neuronal excitability [51,73]. Voltage-gated sodium channels, especially Nav1.7 and Nav1.8, are central to nociceptive signaling because they participate in the initiation and propagation of action potentials in sensory neurons [73]. In TMJ inflammatory pain models, glial interleukin-1β has been shown to upregulate neuronal Nav1.7 in the trigeminal ganglion, contributing to TMJ inflammatory hypernociception [74]. Nav1.8 has also been implicated in inflammatory pain more broadly, although the evidence is not specific to occlusal interference or TMJ inflammation [75]. Therefore, sodium channel regulation should be interpreted as a mechanism of trigeminal nociceptor sensitization.

In addition to sodium channels, potassium channels also influence nociceptor excitability. Kv7/KCNQ channels help stabilize the resting membrane potential and limit repetitive firing in sensory neurons [76]. Inhibition or dysfunction of Kv7/M currents can depolarize nociceptive neurons and promote spontaneous or exaggerated firing, thereby contributing to pain hypersensitivity [77]. Although direct evidence linking Kv7 channels to occlusion-related TMD remains limited, this channel family provides a plausible mechanism by which inflammatory mediators may enhance trigeminal neuronal excitability. Furthermore, acid-sensing ion channels (ASICs) are activated by extracellular acidosis associated with inflammation, ischemia, or tissue injury and can promote sodium influx and nociceptor excitation [78]. In trigeminal ganglion neurons innervating the orofacial region, ASICs have been shown to contribute to orofacial inflammatory pain [79]. Together, sodium channels, Kv7 channels, TRP channels, P2X3 receptors, and ASICs form an ion channel network through which inflammatory and biomechanical inputs may enhance peripheral sensitization in TMD-related pain.

### 4.2. Intracellular Signaling Pathways

Peripheral nociceptor activation and sensitization, as outlined in Section 4.1, do not occur in isolation. Rather, they initiate intracellular signaling events that amplify nociceptive excitability, inflammatory mediator production, and neuroimmune communication. This subsection focuses on three major signaling modules: the mitogen-activated protein kinase (MAPK) pathways [80,81], the phosphoinositide 3-kinase (PI3K)/Akt/mTOR pathway [82,83,84,85], and the cyclic adenosine monophosphate (cAMP)/protein kinase A (PKA)/cAMP response element-binding protein (CREB) pathway [86]. These pathways serve as important nodes in nociceptor sensitization and pain amplification, although their direct contribution to clinical TMD remains incompletely established.

MAPK pathways, including extracellular signal-regulated kinase (ERK), p38 MAPK, and c-Jun N-terminal kinase (JNK), play crucial roles in cellular responses to stress and inflammation [80,87]. In TMD-related pain, inflammatory stimulation of the TMJ can activate MAPK-dependent mechanisms in trigeminal nociceptive pathways. For example, in a rat model, chronic TMJ inflammation and estradiol have been shown to interact through MAPK activation to affect TMJ nociceptive processing by trigeminal caudalis neurons [88]. ERK/CREB signaling is also widely implicated in pain and analgesia, supporting its relevance to activity-dependent neuronal plasticity and nociceptive amplification [86]. p38 MAPK is involved in the production of pro-inflammatory mediators such as TNF-α and IL-1β, which are elevated in inflammatory pain states [80]. In glial cells, MAPK signaling may further promote cytokine production and enhance neuron–glia interactions, thereby linking peripheral inflammation to central sensitization. Thus, MAPK activation should be described as a pain- and inflammation-related signaling mechanism relevant to TMJ nociception.

The PI3K/Akt/mTOR signaling pathway is another important intracellular cascade involved in cell survival, metabolism, protein translation, and neuroplasticity. In pain pathways, PI3K/Akt signaling has been implicated in the development and maintenance of chronic pain, while mTOR-mediated translational control may contribute to persistent nociceptive plasticity [83,84,85]. In joint-related inflammatory conditions, PI3K/Akt/mTOR signaling can regulate inflammatory responses, chondrocyte activity, and cellular stress responses [83]. Although most evidence for this pathway comes from inflammatory pain, osteoarthritis, and neuroplasticity models, it offers a useful framework for understanding how persistent peripheral inflammation may lead to sustained nociceptive signaling. In TMD, PI3K/Akt/mTOR signaling should be considered a candidate pathway for inflammatory and pain-related plasticity, though its exact role needs further validation in TMJ- and trigeminal system-specific studies.

The cAMP/PKA/CREB pathway is pivotal in mediating cellular responses to neurotransmitters, neuropeptides, and inflammatory mediators [86]. This pathway is particularly relevant to CGRP receptor signaling, which can activate adenylyl cyclase, increase intracellular cAMP, and stimulate PKA-dependent downstream responses. By contrast, substance P primarily signals through NK1 receptor-dependent pathways, including PLC/PKC and MAPK signaling, although downstream crosstalk with other intracellular pathways may occur. Activated PKA can phosphorylate downstream targets, including CREB, thereby regulating gene transcription associated with neuronal excitability and pain-related plasticity [86]. In experimentally induced masseter myositis, changes in CGRP levels in muscle and brain tissues further support the involvement of neuropeptide-related signaling in craniofacial muscle pain [89]. Thus, the cAMP/PKA/CREB pathway provides a plausible mechanistic link between CGRP-related signaling, nociceptor sensitization, and longer-term transcriptional responses, although its specific contribution to TMD-related pain requires further validation.

While these intracellular signaling pathways contribute to acute and subacute inflammatory and nociceptive responses, the transition to chronic pain states likely involves more enduring molecular alterations. The following subsection explores how epigenetic modifications may translate transient signaling events into persistent changes in gene expression that sustain neuroinflammation and pain sensitization over the long term.

### 4.3. Epigenetic Changes

The regulation of ion channels and the intracellular signaling cascades discussed above largely mediate acute and subacute phases of nociceptor sensitization and neurogenic inflammation. However, the persistence of pain and inflammation in TMD, even when the initial peripheral input has diminished, suggests the involvement of more enduring molecular mechanisms. Epigenetic modifications, including DNA methylation, histone modifications, and microRNA (miRNA) regulation, can translate transient inflammatory signals into sustained changes in gene expression, thereby contributing to the chronicity of pain and neuroinflammation [90,91].

Epigenetic modifications, particularly DNA methylation and hydroxymethylation, play a crucial role in gene regulation in physiological and pathological contexts, including chronic pain. In chronic TMJ pain, epigenetic regulation of inflammatory mediators has received increasing attention. In a TMJ inflammatory pain model, increased 5-hydroxymethylcytosine (5hmC) at the TNF-α promoter in the trigeminal ganglion was associated with increased TNF-α expression. Moreover, TET1 knockdown in the trigeminal ganglion reversed CFA-induced TNF-α upregulation and alleviated chronic TMJ pain, suggesting that TET1-mediated epigenetic regulation contributes to chronic inflammatory TMJ pain [92]. These findings provide a potential molecular mechanism for the sustained inflammatory signaling and trigeminal sensitization described in Section 3.3.

Histone modifications represent another layer of epigenetic regulation and can significantly influence gene expression and cellular function [93,94]. Histone acetylation is generally associated with transcriptional activation, whereas some histone methylation marks, such as H3K27me3, are associated with transcriptional repression [93]. In chronic pain research, histone acetylation and deacetylation have been implicated in inflammatory signaling, glial activation, and persistent pain states [94]. In TMJ osteoarthritis, emerging evidence suggests that epigenetic biomarkers, including DNA methylation, histone modifications, and non-coding RNAs, may participate in cartilage degradation, inflammation, and disease progression [95]. However, the evidence directly connecting specific histone marks such as H3K9ac or H3K27me3 to TMD-related pain remains limited; therefore, histone modifications should be discussed as emerging mechanisms requiring further validation in TMJ- and TMD-specific models.

**Figure 4:**
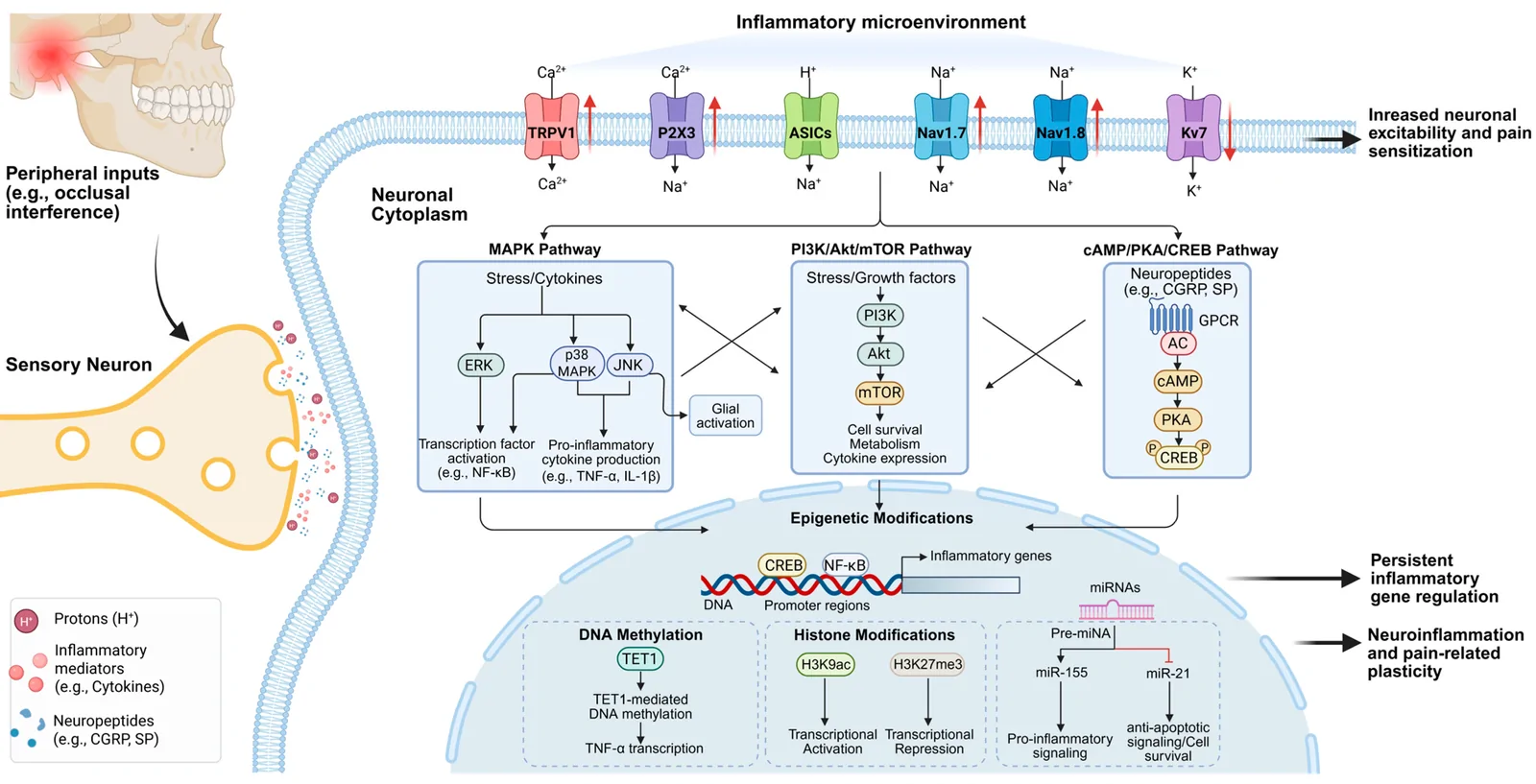
Candidate molecular mechanisms underlying trigeminal nociceptor sensitization and pain-related plasticity. Ion channel regulation, MAPK, PI3K/Akt/mTOR, cAMP/PKA/CREB signaling, and epigenetic regulation may connect peripheral inflammatory or biomechanical inputs to neuronal excitability, inflammatory gene regulation, neuroinflammation, and pain-related plasticity. The arrows next to ion channels indicate inflammation-associated alterations in channel expression or activity. Upward arrows represent increased expression or activation of nociceptive channels (TRPV1, P2X3, ASICs, Nav1.7, and Nav1.8), whereas the downward arrow indicates reduced Kv7 activity associated with neuronal hyperexcitability.

miRNAs are small non-coding RNAs that regulate gene expression post-transcriptionally and may contribute to inflammation, cartilage remodeling, and pain persistence. miR-155 has been implicated in neuropathic pain and neuroinflammation, partly through regulation of inflammatory signaling pathways [96,97]. However, its specific role in TMD or TMJ inflammation requires further confirmation. By contrast, miR-21-5p has been investigated in TMJ osteoarthritis, where it may contribute to cartilage matrix degradation and pathological remodeling [98]. Together, these findings suggest that miRNAs may function as important post-transcriptional regulators of inflammatory signaling and tissue remodeling in TMJ-related disorders, although their direct contribution to TMD pain phenotypes remains an area for future research (Figure 4).

## 5. Pain Sensitization, Behavioral Manifestations, and Sex-Related Differences

The molecular mechanisms discussed in Section 4, including ion channel regulation, intracellular signaling, and epigenetic modulation, ultimately need to be interpreted at the functional level of pain processing. In TMD-related pain, these molecular events may be reflected as peripheral and central sensitization, altered sensory thresholds, spontaneous or evoked pain, emotional and cognitive changes, and sex-related differences in pain processing. This section therefore connects the preceding molecular mechanisms with pain perception, behavioral manifestations, and sex-related differences relevant to TMD.

### 5.1. The Neural Basis of Hyperalgesia

Hyperalgesia, characterized by increased sensitivity to painful stimuli, is rooted in both peripheral and central sensitization mechanisms. Peripheral sensitization occurs when nociceptors become more responsive following exposure to inflammatory mediators at the injury or inflammatory site, including bradykinin, prostaglandins, cytokines, ATP, and neuropeptides [99,100]. Concurrently, central sensitization refers to increased responsiveness of nociceptive neurons within the central nervous system after prolonged or repeated nociceptive input [101,102]. Although an early foundational study provided evidence for a central component of post-injury pain hypersensitivity [101], more recent TMD-specific evidence indicates that patients with TMD may show widespread pressure pain hypersensitivity and enhanced temporal summation, supporting the involvement of central hyperexcitability in at least a subset of patients with TMD [45]. In OPPERA-related research, pressure pain thresholds were also shown to fluctuate with the clinical course of painful TMD, supporting the clinical relevance of pain sensitivity measures while also indicating that they should not be interpreted as simple predictors of disease onset [103]. Collectively, these findings suggest that sensitization-related processes may vary dynamically across the clinical course of TMD-related pain.

Ectopic spontaneous discharges in sensory afferent pathways may also contribute to hyperalgesia and spontaneous pain. Such discharges can arise from peripheral nerve injury or inflammation, leading to abnormal excitability in primary afferent neurons and action potential generation without external stimulation [104]. This activity is facilitated by changes in ion channel expression and function, particularly sodium channel upregulation and potassium channel dysfunction, which enhance neuronal excitability [105,106,107]. In trigeminal pain pathways, similar mechanisms may contribute to persistent orofacial pain and heightened responses to mechanical stimulation [69]. In addition, glial cells, particularly satellite glial cells, astrocytes, and microglia, can modulate neuronal excitability by releasing cytokines and neuromodulators after inflammation or injury [108]. Thus, ectopic activity and glial–neuronal interactions provide plausible mechanisms for spontaneous pain and widespread hypersensitivity, although direct evidence varies across TMD phenotypes.

Pain memory consolidation is another important component of hyperalgesia, whereby previous pain experience influences subsequent pain perception through synaptic plasticity and neural circuit modulation. Long-term potentiation (LTP) and long-term depression (LTD) in spinal and supraspinal pain pathways can alter synaptic strength and contribute to persistent pain amplification [109,110]. Repeated nociceptive stimulation can lead to LTP-like changes in pain pathways, resulting in enhanced synaptic efficacy and heightened pain responses [109]. Moreover, the amygdala and other limbic structures are involved in the affective, emotional, and contextual dimensions of pain memory, which may exacerbate pain perception and contribute to chronic pain persistence [111]. These mechanisms may help explain why pain in some patients persists beyond the initial peripheral stimulus and becomes influenced by emotional and cognitive context.

### 5.2. Pain-Related Behavioral Manifestations

Mechanical allodynia, defined as pain due to a stimulus that does not normally provoke pain, is a feature of various pain conditions, while altered mechanical sensitivity has also been reported in TMD [112,113]. The quantitative assessment of mechanical allodynia and mechanical hyperalgesia can involve standardized methods such as von Frey filament testing in experimental studies, whereas pressure algometry is commonly used to assess pressure pain thresholds in clinical settings. Research indicates that individuals with TMD often exhibit heightened sensitivity to mechanical stimuli in the masticatory and orofacial regions, which can be quantified by pressure pain threshold testing [45,114]. Pressure algometry has also been investigated as a diagnostic and assessment tool in TMD, supporting its value for characterizing sensory dysfunction [114]. Longitudinal evidence further indicates that pressure pain thresholds may fluctuate with the clinical course of painful TMD, although they do not usefully predict disease onset [103]. These findings suggest that mechanical sensitivity measures can help characterize pain severity and sensory dysfunction in TMD, although they should be interpreted together with clinical diagnosis and psychosocial assessment.

Spontaneous pain and pain-related behaviors provide additional insights into the clinical experience of TMD. In patients, these manifestations are more appropriately described as self-reported spontaneous pain, protective jaw use, avoidance of painful movements, impaired chewing, and jaw functional limitation rather than as facial grimacing or vocalization alone. Clinical assessment should therefore combine patient-reported pain intensity, functional limitation, pain-related disability, and psychosocial measures within a multidimensional diagnostic framework [6]. Management-oriented reviews also emphasize that TMD pain assessment should integrate symptom severity, functional impact, and patient-specific psychosocial factors [115]. This approach is more clinically appropriate than relying solely on observational pain behaviors.

Chronic pain conditions such as TMD are often associated with emotional and cognitive changes, including anxiety, depression, catastrophizing, impaired attention, and altered pain coping [6,115,116,117,118]. Research has demonstrated that these emotional and cognitive factors are closely intertwined with pain perception and can exacerbate pain-related disability. In patients with TMD, catastrophizing has been associated with symptom severity and poorer treatment outcomes, and assessing catastrophizing may therefore be valuable in clinical management [117]. Neuroimaging studies have also reported altered brain structure and function in TMD-related pain, including abnormalities during cognitive and emotional tasks [119,120,121]. Interventions such as cognitive–behavioral therapy (CBT), which target emotional and cognitive factors, show promise in improving pain, pain-related interference, and coping in patients with TMD [118,122]. Integrating psychological support into TMD management therefore helps address the emotional and cognitive dimensions of chronic pain.

### 5.3. Sex Differences in Pain

Estrogen receptor signaling is important for understanding sex differences in pain, as estrogen can modulate nociceptive signaling, pain sensitivity, and analgesic responses [123,124]. Fluctuations in estrogen across the menstrual cycle may influence pain perception, although the direction and magnitude of this effect can vary across pain modalities and patient populations [125]. The presence of estrogen receptors in pain-processing regions of the nervous system further suggests that hormonal fluctuations may contribute to sex-related variability in pain sensitivity and pain modulation [123,124].

Neuroimmune regulation adds further complexity to sex differences in pain. Recent, largely preclinical work suggests that immune and glial contributions to pain hypersensitivity may differ between males and females, involving interactions among sex hormones, immune cells, microglia, T cells, and cytokine signaling [126,127]. For example, preclinical studies indicate that different immune cell populations can mediate mechanical pain hypersensitivity in male and female animals [127]. Therefore, sex differences in TMD-related pain should not be attributed only to hormone levels but should also be considered within a broader neuroimmune and biopsychosocial framework [124,126,128].

The neural circuitry involved in pain processing may also show sex-related differences, influencing how pain is perceived, evaluated, and modulated [128,129]. Neuroimaging studies in experimental pain have reported sex differences in activation of pain-related regions, including the medial prefrontal cortex, anterior cingulate cortex, insula, thalamus, and midcingulate cortex [129,130,131]. However, such findings should be interpreted cautiously because they are influenced by stimulus modality, study design, hormonal status, psychological factors, and sociocultural context [124,128]. Recognizing sex-related differences across molecular, neuroimmune, and systems-level pain mechanisms may support more individualized approaches to TMD pain assessment and management (Table 1).

## 6. Translational Perspectives and Current Clinical Limitations

### 6.1. Mechanism-Oriented Animal Models

Animal models that reproduce selected peripheral biomechanical or inflammatory inputs relevant to TMD are useful for dissecting muscle–nerve signaling mechanisms and for preclinical testing of candidate interventions [132,133]. Rodent models have been developed by altering occlusal conditions, inducing TMJ inflammation, modifying mandibular loading, or using devices that disturb jaw function. These models may reproduce selected features relevant to TMD research, such as mechanical hypersensitivity, altered jaw function, reduced bite force, TMJ inflammation, and masticatory muscle-related pain behaviors [132,134]. However, they should be interpreted as models of specific mechanisms or phenotypes rather than as complete replicas of clinical TMD.

To validate whether these models engage mechanisms relevant to peripheral sensitization, neurogenic inflammation, and central sensitization, a multimodal phenotyping approach is required. First, standardized behavioral assessments are important for quantifying pain-related outcomes. Commonly employed methods include von Frey filament testing to measure mechanical allodynia, bite force to evaluate functional impairment, and feeding or orofacial behavior analysis to capture changes in spontaneous jaw use [134,135]. These behavioral readouts provide a translational link to the hyperalgesia, allodynia, and functional limitation mechanisms described in Section 5.

Second, histological and biochemical analyses of trigeminal ganglia, spinal trigeminal nucleus, TMJ tissues, synovium, and masticatory muscles can be used to evaluate receptor expression, neuropeptide release, inflammatory mediators, and glial activation. Examples include TRPV1/TRPV4/P2X3-related nociceptive signaling, CGRP and substance P expression, cytokine changes, and GFAP/Iba1 upregulation in trigeminal pain pathways [69,132,133,136]. Third, advanced MRI-based structural, functional, and diffusion approaches may support longitudinal research phenotyping of tissue remodeling and pain-related alterations in TMD [121]. By contrast, TSPO-PET remains an exploratory neuroinflammation imaging tool, and its TMD-specific translational validity has not yet been established [137,138,139]. Integration of behavioral, tissue-level, molecular, and imaging readouts may improve the mechanistic validity of animal models while avoiding overextension from preclinical findings to clinical causality (Table 2).

### 6.2. Biomarkers Targeting Neuroinflammatory Pathways

Translating mechanistic insights into clinical practice requires biomarkers that are accessible, reproducible, and clinically interpretable. Based on the signaling cascades detailed in Section 3 and Section 4, including TRP/P2X3 activation, CGRP/substance P release, cytokine signaling, and glial responses, candidate biomarkers may help stratify TMD phenotypes and monitor treatment responses [148,149]. At present, however, most biomarkers in TMD remain exploratory rather than clinically established.

Salivary biomarker assessment provides a non-invasive peripheral approach for evaluating inflammatory, stress-related, and pain-related mediators in patients with TMD [150,151]. Salivary cytokines, cortisol, oxidative stress markers, and pain-related molecules have been investigated in TMD, but the literature remains heterogeneous, and the diagnostic specificity of individual markers is limited [148,150,151]. Therefore, salivary profiling should be described as a promising research tool rather than a real-time readout of neurogenic inflammation. Neuropeptides such as CGRP and substance P are biologically relevant to trigeminal neurogenic inflammation, but evidence supporting salivary CGRP or substance P as validated TMD biomarkers remains insufficient. Current evidence more strongly supports their mechanistic relevance in trigeminal pain pathways than their immediate clinical utility as diagnostic tests [152,153].

Blood or plasma biomarkers provide a minimally invasive window into systemic inflammatory and neuroimmune status. Cytokines such as IL-1β, TNF-α, IL-6, and IL-8, as well as stress-related and oxidative markers, have been examined in TMD and other orofacial pain conditions [148,149]. Plasma CGRP has also been evaluated across chronic TMD, migraine, and combined TMD–migraine phenotypes; however, interictal plasma concentrations did not differ significantly among these groups, indicating limited diagnostic utility in that study [154]. These negative biomarker findings do not exclude the possible mechanistic relevance of CGRP-related signaling in trigeminal pain comorbidity [152,154]. Nevertheless, blood biomarkers are generally less specific for TMJ or masticatory muscle pathology than local synovial or tissue-based measures, and their interpretation can be influenced by systemic inflammation, stress, medication use, comorbid pain, and sex-related biological variability.

Cerebrospinal fluid (CSF) and neuroimaging biomarkers represent higher-complexity approaches for studying central sensitization and neuroinflammation. At present, CSF biomarkers should not be presented as clinically established markers for TMD because TMD-specific clinical validation remains insufficient [148,149]. Similarly, TSPO-PET has been used to investigate neuroinflammation in several neurological conditions [137], but direct clinical validation in TMD remains lacking [148,149]. MRI-based techniques, including DTI, may help characterize structural or functional alterations in pain-related brain and trigeminal pathways, but these approaches are currently better suited to research phenotyping than routine clinical staging [121]. Taken together, biomarker research in TMD is most valuable at present for refining pain phenotypes and testing mechanistic hypotheses, whereas its routine clinical application still requires longitudinal validation and clearer links to treatment response.

### 6.3. Mechanism-Informed Interventions and Current Standard of Care

The mechanisms discussed in this review may inform future therapeutic strategies, but they should be interpreted within the current standard of care for TMD. At present, most TMD patients are managed initially with conservative and reversible approaches, including patient education, self-management, behavioral modification, physical therapy, and pharmacotherapy, together with the provisional and time-limited use of occlusal appliances when appropriately indicated [4,9]. Therefore, mechanism-based interventions should be framed as complementary or future directions rather than replacements for established conservative management.

One potential translational direction is to reduce peripheral nociceptor hyperexcitability. Ion channels and receptors such as TRPV1/TRPV4, P2X3, Nav1.7, and Nav1.8 are mechanistically relevant to trigeminal nociceptive signaling, but most pharmacological evidence remains preclinical or extrapolated from other pain conditions. For example, sensory neuron TRPV4 has been implicated in TMJ pain models through modulation of CGRP release, supporting its relevance as a research target [19]. However, selective ion channel blockers have not yet become established TMD-specific treatments. Botulinum toxin A has been investigated for TMD-related myalgia and masseter hyperactivity, but the clinical evidence is mixed: it is better supported for masseter hypertrophy and remains equivocal for myogenous TMD, with little evidence for intra-articular TMJ disorders [155]. Overall, these targets are useful for defining peripheral pain-generating mechanisms, but their therapeutic relevance in TMD will depend on identifying the specific patient subgroups in which peripheral nociceptor hyperexcitability is a dominant driver of symptoms.

A second translational direction involves neuropeptide-mediated neurovascular signaling. CGRP-targeting therapies have achieved successful clinical translation in migraine [156,157], and CGRP-related mechanisms may be relevant to trigeminal pain biology and TMD–headache comorbidity [152], although interictal plasma CGRP did not distinguish TMD, migraine, and combined TMD–migraine phenotypes in one clinical study [154]. However, direct evidence for gepants or anti-CGRP monoclonal antibodies in TMD is currently insufficient. Therefore, CGRP-targeted therapy should be presented as a hypothesis-generating direction for selected comorbid phenotypes rather than an available TMD treatment strategy. Substance P/NK1 signaling has stronger preclinical relevance to TMJ inflammation, as NK1 receptor activation in trigeminal ganglion neurons contributes to mechanical allodynia in rat TMJ inflammation models [158]. Nevertheless, translation of NK1 antagonism to clinical TMD treatment remains to be established.

At the central level, modulation of central sensitization and glial activation remains an important mechanistic direction. MAPK signaling, microglial activation, and neuron–glia interactions have been implicated in chronic pain models and in mechanistic accounts of painful TMD [159,160,161]. However, pharmacological inhibitors of p38 MAPK, JNK, or glial activation are not currently standard treatments for TMD. Non-pharmacological approaches, especially physical therapy, exercise-based rehabilitation, behavioral interventions, and neuromodulatory modalities, may help reduce pain and improve function through multimodal mechanisms. TENS and other electrical stimulation modalities have shown potential for pain reduction in TMD, although the certainty of evidence and optimal protocols vary across studies [162]. Their translational value may lie less in targeting a single molecular pathway than in providing multimodal symptom control, particularly pain reduction, while their effects on jaw function and other patient-centered outcomes require further study.

Occlusal interventions require particular caution. The current evidence does not support irreversible occlusal adjustment as a routine treatment for TMD, and an earlier systematic review concluded that occlusal adjustment cannot be recommended for treating or preventing TMD [163]. A more recent systematic review also reported uncertain or inconclusive evidence for many occlusal appliances and interventions [164]. In line with contemporary standard-of-care recommendations, occlusal adjustment or restorative occlusal modification should be reserved for clearly indicated dental situations, such as acute iatrogenic occlusal changes or independent restorative needs, rather than used as a general strategy to eliminate a presumed nociceptive root cause [9]. This distinction is essential for avoiding overinterpretation of mechanistic models as clinical causality.

In summary, translational research in TMD should proceed from mechanism-informed phenotyping toward conservative, reversible, and patient-centered management. Molecular targets such as ion channels, CGRP/substance P signaling, MAPK pathways, and glial activation provide valuable directions for future research, but clinical treatment decisions should remain grounded in validated diagnosis, pain phenotype, psychosocial assessment, functional limitation, and current evidence-based standards of care.

## 7. Research Challenges and Future Directions

### 7.1. Limitations of Current Mechanistic Studies

Despite the substantial progress summarized in Section 3, Section 4, Section 5 and Section 6, ranging from peripheral nociceptor sensitization to neurogenic inflammation, glial activation, and pain-related plasticity, several critical gaps continue to limit mechanistic interpretation and translational predictability.

A major limitation concerns the incomplete recapitulation of chronic, multifactorial human TMD by existing animal models. Many rodent studies use acute or subacute occlusal alteration, chemically induced TMJ inflammation, surgical injury, or other single-factor paradigms [132,140,165]. These models are valuable for isolating specific mechanisms, such as peripheral sensitization, inflammatory mediator release, joint degeneration, or trigeminal pathway activation, but they cannot fully reproduce the biological, psychological, behavioral, and environmental interactions that shape clinical TMD [132,165,166]. This limitation is particularly important because painful TMD often develops within a broader biopsychosocial context involving pain sensitivity, stress, anxiety, sleep disturbance, parafunctional behaviors, and individual susceptibility [7,167]. Therefore, animal models should be interpreted as tools for testing defined mechanisms or phenotypes rather than as complete replicas of human TMD.

Model construction related to occlusal alteration requires particular caution. Experimental occlusal interference, resin elevation, unilateral anterior crossbite, or other dental modifications may impose abrupt and relatively large biomechanical perturbations that do not necessarily correspond to those typically encountered in clinical settings [132,140]. In addition, rodents and humans differ in TMJ anatomy, occlusal biomechanics, craniofacial growth, and pain expression [165,168]. As a result, the same experimental intervention may produce a traumatic or degenerative response in animals that should not be directly equated with clinical occlusal interference. These differences do not invalidate animal models, but they require careful alignment between the model, the mechanistic question, and the intended clinical inference.

A further limitation stems from species differences in molecular signaling. The biophysical properties and expression patterns of several nociception-related ion channels, including Nav1.7, Nav1.8, and TRPV1, may differ between rodents and humans. Human DRG studies have shown distinct electrophysiological properties of human sensory neurons, and human Nav1.8 exhibits persistent and ramp currents that may contribute to species-specific firing patterns [169,170,171,172,173]. These findings underscore the need for caution when extrapolating drug effects from rodent nociceptors to human trigeminal pain conditions. Human tissue, human sensory neuron models, and cross-species validation will therefore be important for improving the translational relevance of molecular targets.

Another critical gap is the static assessment of dynamic peripheral–central interactions. Many studies measure inflammatory mediators, neuropeptides, ion channels, or glial markers at a single endpoint, which limits the ability to determine temporal relationships among peripheral inflammation, trigeminal ganglion activation, and central sensitization [132,165,166]. Recent clinical evidence indicates that patients with chronic TMD may show a greater propensity to develop experimentally induced secondary mechanical hyperalgesia, a proxy for central sensitization [174], but the sequence by which peripheral input becomes coupled to central pain amplification remains insufficiently defined. Longitudinal, multi-compartment sampling strategies are needed to determine whether changes such as CGRP elevation, cytokine upregulation, satellite glial cell activation, or Cx43 remodeling precede, accompany, or follow the development of persistent pain.

Equally important, multi-system interactions remain underrepresented in mechanistic studies. The TMJ does not function in isolation; it is embedded within a network involving the masticatory muscles, cervical region, autonomic and stress-regulatory systems, sleep regulation, and psychosocial factors [7,175]. Mechanistic studies often isolate the trigeminal pathway to reduce experimental complexity, but this approach may overlook interactions among muscle function, stress physiology, immune responses, and central pain modulation. Future models should therefore integrate neural, immune, vascular, behavioral, and psychosocial readouts rather than relying on a single molecular or anatomical endpoint [165].

Addressing these limitations will require longer-term and phenotype-specific animal models, cross-species validation of key molecular targets, human-derived experimental platforms, longitudinal multi-compartment sampling, and systems-level designs that connect peripheral tissue events with central pain processing and clinical symptoms.

### 7.2. Technical Innovation Needs

#### 7.2.1. High-Spatiotemporal-Resolution Imaging

To move beyond static histology and endpoint biochemistry, advanced imaging technologies are needed to visualize the dynamics of muscle–nerve, neurovascular, and immune interactions. Intravital two-photon microscopy provides a peer-reviewed methodological basis for imaging cellular activity and blood flow dynamics in living tissues [176,177,178]. In TMD-related research, this approach could be adapted to examine how trigeminal sensory terminals, local vascular responses, and immune cell behavior change after controlled inflammatory or biomechanical perturbation. For example, combining genetically encoded calcium indicators in sensory neurons with vascular tracers or immune-cell reporters may allow time-resolved visualization of neurovascular coupling and inflammatory amplification [177,178]. These applications should be framed as future experimental possibilities rather than as techniques already validated for occlusion-related TMD.

Super-resolution microscopy, such as STED or PALM, may further resolve nanoscale changes in sensory neuron architecture, ion channel localization, and neuron–glia interfaces [179,180]. This is relevant because sensory neurons can contain an axon initial segment associated with spontaneous activity in neuropathic pain [181], and inflammatory mediators can influence Nav1.7 delivery and distribution along sensory axons [182]. Such approaches may help test whether persistent inflammatory or mechanical input changes the spatial organization of voltage-gated sodium channels, receptor clusters, or satellite glial cell processes in trigeminal ganglia. Finally, PET imaging with TSPO ligands remains a promising method for studying neuroinflammation, but in the context of TMD, it should currently be regarded as a research tool rather than a clinically validated biomarker of neuroinflammation or central pain mechanisms in TMD [138,139,183].

#### 7.2.2. Single-Cell and Spatial Omics

The cellular heterogeneity within the TMJ, masticatory muscles, trigeminal ganglion, and associated immune microenvironments remains incompletely understood. Single-cell RNA sequencing can help resolve this complexity by identifying transcriptionally distinct sensory neurons, satellite glial cells, macrophages, fibroblasts, chondrocytes, endothelial cells, and immune cell populations [184,185,186,187,188]. In trigeminal research, scRNA-seq has already been used to characterize dental sensory and proprioceptive trigeminal neurons [184], providing a foundation for future studies of TMJ- and masticatory muscle-related afferents. In TMJ osteoarthritis models induced by different occlusal disorder paradigms, single-cell transcriptomic analysis has also revealed chondrocyte heterogeneity and immune–chondrocyte crosstalk, illustrating how model-specific mechanisms may differ even within occlusion-related experimental systems [187].

For pain mechanisms, single-cell approaches may help identify nociceptor subsets enriched for CGRP, P2X3, TRPV1, or sodium-channel signatures, as well as satellite glial cell states associated with GFAP, cytokine, or Cx43-related signaling [186,189,190,191]. Single-cell or single-nucleus ATAC-seq could further define cell-type-specific chromatin accessibility and regulatory programs associated with inflammatory pain [192]. However, dissociation-based single-cell approaches lose spatial information. Spatial transcriptomics, including platforms such as Visium, MERFISH, or other spatially resolved methods, can preserve tissue architecture and help map how nociceptor terminals, vascular cells, immune cells, fibroblasts, and synovial structures are organized within TMJ tissues [188,193]. These approaches are especially useful for generating hypotheses about cell–cell communication, but inferred ligand–receptor interactions should be validated by protein-level and functional experiments before being interpreted as therapeutic targets.

#### 7.2.3. Human Microphysiological Systems

Traditional animal models cannot fully replicate human TMJ anatomy, biomechanics, immune signaling, or nociceptor biology. Human microphysiological systems, including organ-on-a-chip and joint-on-a-chip platforms, offer a complementary approach for modeling human tissue interactions under controlled mechanical and biochemical conditions [194,195,196]. A future TMJ-oriented neurovascular microphysiological system could incorporate human iPSC-derived sensory neurons, endothelial cells, synovial fibroblasts, chondrocytes, macrophages, and extracellular matrix components within a mechanically tunable microfluidic environment. Such platforms could be used to test how cyclic compression, shear stress, inflammatory cytokines, or neuropeptides influence nociceptor activation, endothelial permeability, cytokine release, and tissue remodeling.

These systems should not be presented as immediate replacements for animal models or clinical studies. Their value lies in providing controllable human-relevant platforms for testing specific hypotheses, such as neuron–synovium communication, neurovascular permeability, or patient-specific responses to ion channel modulators and anti-inflammatory agents. Patient-specific iPSC-derived sensory neurons may help model genetic variability in pain-related pathways, particularly SCN9A-related pain disorders [197,198]. More broadly, human pluripotent stem cell-derived sensory neurons can express TAC1, SCN9A, and SCN10A and can support TRPV1-targeted pharmacological testing [199,200]. Compared with static two-dimensional cell cultures, microphysiological systems can better incorporate flow, mechanical force, and multicellular crosstalk. Their future contribution will depend on standardization, reproducibility, validation against human tissue data, and integration with clinically meaningful pain phenotypes.

### 7.3. Translational Medicine Prospects

The principal challenge for TMD translational research is not simply to identify additional molecular mechanisms but to determine which mechanisms are clinically relevant in which patient subgroups. Current TMD management remains grounded in conservative and reversible care, including patient education, self-management, physical therapy, behavioral interventions, and pharmacotherapy, with provisional and time-limited use of occlusal appliances when appropriately indicated [4,9]. Mechanism-based targets such as ion channels, CGRP/substance P signaling, glial activation, MAPK pathways, and epigenetic regulators should therefore be viewed as future directions for phenotype refinement and therapeutic development rather than as replacements for established care.

A key priority is to develop clinically usable stratification frameworks. Rather than assuming that all patients follow a single peripheral-to-central trajectory, future research should identify reproducible subgroups characterized by dominant peripheral nociceptive input, inflammatory joint pathology, myofascial pain, psychosocial vulnerability, central sensitization-related features, or overlapping headache and widespread pain phenotypes [6,201,202]. Biomarkers, quantitative sensory testing, neuroimaging, and psychosocial assessment may contribute to such stratification [6,45,121,148,149,201,202], but their value will depend on whether they predict symptom trajectories, treatment response, or risk of chronicity in longitudinal cohorts. Thus, the field should move gradually from exploratory biomarker discovery toward validated, clinically interpretable phenotyping.

Future clinical trials should also reflect biological and psychosocial heterogeneity. Conventional trials that enroll broadly defined TMD populations may obscure treatment effects because different pain mechanisms are mixed within the same study population [203]. Enrichment strategies based on pain phenotype, sensory profile, inflammatory markers, or comorbid headache may improve signal detection, but these strategies require prior validation [204,205]. Pharmacodynamic endpoints, patient-reported outcomes, functional measures, and long-term follow-up should be integrated so that mechanistic changes can be linked to clinically meaningful improvement.

Neuromodulatory approaches also deserve further investigation, particularly for patients with persistent pain and central sensitization-related features. Non-invasive brain stimulation techniques such as repetitive transcranial magnetic stimulation and transcranial direct current stimulation have been explored in chronic orofacial pain, including TMD, and appear promising but remain limited by small sample sizes, heterogeneous protocols, and risk of bias [206,207,208]. Their future role may lie in improving pain modulation, pain-related interference, and function in selected patients, although any preferential benefit in phenotypes with central sensitization-related features remains to be tested prospectively.

Looking ahead, integration of multi-omics, advanced imaging, quantitative sensory testing, ecological momentary assessment of symptoms and behaviors, and computational modeling may help refine TMD phenotyping [209]. Machine learning approaches could eventually support prediction of disease trajectories and treatment response, but only if based on high-quality, harmonized, longitudinal data [210,211]. Future translational work should therefore prioritize longitudinal validation, standardized phenotyping, and treatment-response endpoints so that proposed molecular and imaging markers can be tested against clinically meaningful outcomes rather than remaining isolated mechanistic observations.

## 8. Conclusions

TMD is a multifactorial condition shaped by biological, psychological, behavioral, and environmental factors. Within this broader framework, peripheral biomechanical inputs, including occlusal interference in selected contexts, may contribute to trigeminal nociceptor activation, neuropeptide release, neurogenic inflammation, glial responses, and pain sensitization. This review therefore does not re-establish occlusal interference as a primary etiological determinant of TMD but rather positions it as one of several potential peripheral inputs that may interact with muscle–nerve signaling and neuroimmune mechanisms.

The evidence summarized in this review supports the biological plausibility of several mechanistic pathways, including TRP channel and P2X3 receptor activation, Nav channel-mediated nociceptor excitability, CGRP/substance P signaling, MAPK- and PI3K/Akt/mTOR-related plasticity, glial activation, and epigenetic regulation. However, the strength of evidence varies substantially across human studies, animal models, and in vitro systems. Preclinical findings provide important mechanistic insight, but they should not be directly equated with clinical causality, especially given the heterogeneity of TMD phenotypes and the inconsistent clinical association between occlusal factors and TMD.

From a clinical perspective, current TMD management should remain grounded in conservative, reversible, and patient-centered approaches, including education, self-management, physical therapy, behavioral interventions, and pharmacotherapy, with provisional and time-limited use of occlusal appliances when appropriately indicated. Mechanism-based targets such as ion channels, neuropeptide signaling, glial modulation, and epigenetic regulation are promising directions for future research, but they have not yet replaced established standard-of-care approaches in routine clinical practice.

Future progress will depend on connecting mechanistic hypotheses with clinically meaningful phenotypes. Longitudinal cohorts, human-derived models, single-cell and spatial omics, dynamic imaging, quantitative sensory testing, and mechanism-informed clinical trials will be needed to determine which patients are most likely to develop persistent pain following peripheral or other nociceptive inputs, which pathways may predominate at different disease stages, and which interventions can modify these trajectories. A cautious, evidence-based integration of mechanistic insight with clinical phenotyping may ultimately support more precise and individualized management of TMD.

## Figures and Tables

**Table 1:** Summary of Pain Perception and Behavioral Changes in TMD.

Category	Subcategory	Key Components	Core Mechanisms/Features	Assessment/Indicators	Clinical/Research Relevance	Key References
Neural Basis of Hyperalgesia	Peripheral sensitization	Nociceptors, inflammatory mediators, TRP channels, sodium channels	Enhanced responsiveness of primary afferent neurons; inflammatory mediator-induced nociceptor sensitization	Mechanical/thermal pain thresholds; pressure pain threshold	Initiates pain amplification and may interact with central sensitization	[48,49,50,51,74,99,100,114]
	Central sensitization	Spinal trigeminal nucleus/trigeminal subnucleus caudalis, thalamus, cortex	Increased central neuronal excitability; enhanced temporal summation; allodynia and widespread pressure pain sensitivity	Quantitative sensory testing; temporal summation; functional imaging	Contributes to chronic TMD pain and widespread hypersensitivity	[45,101,102,121]
	Ectopic spontaneous discharges	Trigeminal ganglion, primary afferent neurons, sodium channels	Abnormal action potential generation without external stimulation; altered ion channel expression	Electrophysiological recording in experimental models	May contribute to spontaneous pain and persistent hypersensitivity	[104,105,106,107]
	Pain memory consolidation	Amygdala, limbic structures, spinal/trigeminal pain pathways	LTP-like plasticity; emotional and contextual modulation of pain	Behavioral conditioning; neuroimaging; pain-related emotional assessment	May contribute to maladaptive pain persistence	[109,110,111]
Pain-Related Behavioral Manifestations	Mechanical allodynia/mechanical hyperalgesia	Masticatory and orofacial regions	Pain or heightened sensitivity evoked by mechanical stimuli	von Frey filament testing; pressure algometry; pressure pain threshold	Helps characterize sensory dysfunction and monitor clinical change	[45,103,112,113,114]
	Spontaneous pain and functional limitation	Jaw function, chewing, protective jaw use, pain-related disability	Ongoing pain, avoidance of painful movement, impaired jaw function	Pain intensity scales; Jaw Functional Limitation Scale; DC/TMD Axis II tools	Supports multidimensional clinical assessment	[6,115]
	Emotional and cognitive changes	Anxiety, depression, catastrophizing, attention, coping	Psychological factors interact with pain perception and disability	Psychological scales; cognitive–behavioral assessment; neuroimaging	Highlights need for multidisciplinary management, including CBT	[116,117,118,120,121,122]
Sex Differences in Pain	Estrogen receptor signaling	Estrogen, ERα, ERβ	Hormonal modulation of nociceptive signaling and pain sensitivity	Hormonal assays; menstrual cycle phase; receptor expression studies	Informs sex-aware pain assessment	[123,124,125]
	Neuroimmune regulation	Microglia, T cells, cytokines, immune cells	Sex-dependent immune and glial contributions to pain hypersensitivity	Cytokine profiling; glial activation markers; experimental immune assays	May help explain sex-related variability in pain mechanisms	[126,127,128]
	Neural circuit dimorphism	Prefrontal cortex, anterior cingulate cortex, insula, thalamus, midcingulate cortex	Sex-related differences in pain-related brain activation and modulation	fMRI, PET, experimental pain paradigms	Supports individualized pain management and sex-aware research design	[128,129,130,131]

**Table 2:** Mechanism-oriented animal models for investigating TMD-related peripheral inputs, pain sensitization, and neuroinflammatory mechanisms.

Model Category	Mechanistic Target	Model Induction Method	Key Phenotypes	Research Applications	Key References
Experimental occlusal alteration model	Abnormal mechanical loading; peripheral nociceptor sensitization; muscle–joint remodeling	Unilateral or bilateral resin elevation, bonded/metallic crowns, unilateral anterior crossbite, or other dental modifications	TMJ inflammatory or degenerative changes; masticatory muscle hyperalgesia and remodeling; altered trigeminal P2X3-related signaling	Mechanotransduction; muscle–nerve and joint interactions; selected sensitization mechanisms	[23,43,44,49,132,133,140]
Complete Freund’s Adjuvant (CFA) model	Peripheral inflammation; neurogenic inflammation; trigeminal neuroimmune activation	Intra-articular or periarticular CFA administration to the TMJ	Persistent inflammation and mechanical hypersensitivity; altered CGRP/substance P signaling; satellite glial cell and microglial activation	Inflammatory pain; neuropeptide signaling; neuron–glia and neuroimmune interactions	[11,16,48,74,132,133]
Monosodium iodoacetate (MIA) model	Cartilage degradation; subchondral bone remodeling; osteoarthritis-like pathology	Intra-articular MIA injection into the TMJ	Time-dependent synovitis, cartilage loss, subchondral bone erosion and sclerosis, osteophyte formation, and pain-related behavior	TMJ osteoarthritis; cartilage–bone interactions; osteoarthritic pain mechanisms	[132,133,141]
Trigeminal nerve injury model, e.g., IAN transection or ligation	Trigeminal neuropathic pain; ectopic afferent activity; central plasticity	Inferior alveolar nerve transection, ligation, or chronic constriction injury	Facial mechanical allodynia; spontaneous or ectopic afferent activity; altered sodium and potassium currents; trigeminal central plasticity	Trigeminal neuropathic orofacial pain mechanisms; not a direct model of common TMD	[17,66,69,104,133,135,142]
Repetitive jaw loading/overuse model	Mechanical overload; TMJ pain; cartilage remodeling; muscle–joint interaction	Repeated forced mouth opening, controlled jaw loading, or overuse paradigms	TMJ mechanical hypersensitivity; cartilage thinning and remodeling; osteoarthritis-like structural changes; altered jaw function	Biomechanical overloading; joint pain; early degenerative mechanisms	[132,133,143]
Psychological stress model	HPA-axis activation; stress-related modulation of pain	Acute or chronic restraint stress, unpredictable stress, or stress combined with a TMJ nociceptive challenge	Altered corticosterone/ACTH levels and TMJ nociceptive responses; stress-related hyperalgesia; anxiety-like behavior depending on protocol	Stress-related modulation of orofacial and TMJ pain; neuroendocrine–pain interactions	[132,133,144]
Estrogen modulation model	Hormonal regulation; sex-related differences in pain	Ovariectomy with or without estrogen replacement, estrous-cycle manipulation, or sex comparisons	Altered TMJ nociceptive processing; sex-dependent pain responses; context-dependent inflammatory and MAPK signaling	Sex dimorphism; hormone–pain and hormone–inflammation interactions	[88,123,124,125,126,127,128,132,133]
Genetic/transgenic models	Specific molecular pathways, such as TRPV4–CGRP signaling	Global or conditional knockout and transgenic manipulation of candidate pathways, e.g., sensory neuron-specific Trpv4 deletion	Altered nociception, neuropeptide release, inflammatory responses, or neuronal sensitization	Causal mechanistic validation; molecular target prioritization; preclinical therapeutic testing	[19,51,132,136]
Acidic saline masseter model	Persistent myalgia; altered muscle-afferent excitability; sensitization-related mechanisms	Repeated intramuscular acidic saline injections into the masseter muscle	Persistent bilateral mechanical hypersensitivity and altered muscle-spindle afferent excitability in some protocols; results are protocol-dependent	Persistent craniofacial myalgia and sensitization-related mechanisms; not a complete TMD or fibromyalgia model	[133,135,145,146]
Disk displacement/mechanical injury model	Internal derangement; joint structural disruption; secondary osteoarthritis-like remodeling	Surgical anterior disk displacement or controlled joint injury	Persistent disk displacement; fibrocartilage and subchondral bone remodeling; osteophyte formation; inflammatory or degenerative changes	TMJ internal derangement; structural pathogenesis; joint biomechanics and secondary TMJ osteoarthritis	[132,133,147]

## Data Availability

No new data were created or analyzed in this study. Data sharing is not applicable to this article.

## Abbreviations

The following abbreviations are used in this manuscript:

TMD	Temporomandibular disorders
TMJ	Temporomandibular joint
CGRP	Calcitonin gene-related peptide
TRP	Transient receptor potential
TRPV1	Transient receptor potential vanilloid 1
TRPV4	Transient receptor potential vanilloid 4
GTOs	Golgi tendon organs
NPY	Neuropeptide Y
VIP	Vasoactive intestinal peptide
NO	Nitric oxide
VEGF	Vascular endothelial growth factor
GFAP	Glial fibrillary acidic protein
NGF	Nerve growth factor
BDNF	Brain-derived neurotrophic factor
TLR4	Toll-like receptor 4
NF-κB	Nuclear factor kappa B
Cx43	Connexin 43
Nav1.7	Voltage-gated sodium channel Nav1.7
Nav1.8	Voltage-gated sodium channel Nav1.8
Kv7	Voltage-gated potassium channel Kv7
ASICs	Acid-sensing ion channels
MAPK	Mitogen-activated protein kinase
ERK	Extracellular signal-regulated kinase
JNK	c-Jun N-terminal kinase
PI3K	Phosphoinositide 3-kinase
mTOR	Mammalian target of rapamycin
cAMP	Cyclic adenosine monophosphate
PKA	Protein kinase A
CREB	cAMP response element-binding protein
miRNA	microRNA
5hmC	5-hydroxymethylcytosine
TET1	Ten-eleven translocation methylcytosine dioxygenase 1
H3K9ac	Histone H3 lysine 9 acetylation
H3K27me3	Histone H3 lysine 27 trimethylation
LTP	Long-term potentiation
LTD	Long-term depression
CNS	Central nervous system
CBT	Cognitive–behavioral therapy
ERα	Estrogen receptor alpha
ERβ	Estrogen receptor beta
CFA	Complete Freund’s Adjuvant
MIA	Monosodium iodoacetate
IAN	Inferior alveolar nerve
HPA	Hypothalamic–pituitary–adrenal
PET	Positron emission tomography
TSPO	Translocator protein
MRS	Magnetic resonance spectroscopy
DTI	Diffusion tensor imaging
DAMPs	Damage-associated molecular patterns
NfL	Neurofilament light chain
NK1	Neurokinin 1 receptor
TENS	Transcutaneous electrical nerve stimulation
scRNA-seq	Single-cell RNA sequencing
ATAC-seq	Assay for transposase-accessible chromatin sequencing
MPS	Microphysiological systems
iPSC	Induced pluripotent stem cell
STED	Stimulated emission depletion
PALM	Photoactivated localization microscopy
TNF-α	Tumor necrosis factor alpha
IL-1β	Interleukin-1 beta
IL-6	Interleukin-6
ATP	Adenosine triphosphate
